# Multimodal PET, Diffusion Tensor Imaging, and Volumetric MRI Findings in Orbitofrontal Cortex Dysfunction: A Quantitative Single-Case Analysis

**DOI:** 10.7759/cureus.107300

**Published:** 2026-04-18

**Authors:** Jorge A. Pineda, Ryan A Shahrokni, Mohamed F Dounane, Zennen Dellalonga, Joseph C Wu

**Affiliations:** 1 Department of Psychiatry &amp; Human Behavior, School of Population &amp; Public Health, University of California, Irvine, USA; 2 Department of Psychiatry &amp; Human Behavior, School of Biological Sciences, University of California, Irvine, USA; 3 Department of Psychiatry &amp; Human Behavior, School of Biological Sciences, University of California, San Diego, USA; 4 Department of Psychiatry &amp; Human Behavior, School of Medicine, University of California, Irvine, USA

**Keywords:** adverse childhood experiences, fetal alcohol spectrum disorder (fasd), impulse control deficits, orbitofrontal cortex syndrome, traumatic brain injury

## Abstract

Fetal alcohol spectrum disorder (FASD) is a neurodevelopmental condition associated with executive dysfunction and impaired impulse control. When compounded by traumatic brain injury (TBI) and adverse childhood experiences (ACEs), FASD may substantially elevate the risk of violent behavior. This case study examines a 35-year-old violent offender with confirmed FASD, multiple TBIs, and a high ACE score, using multimodal neuroimaging to explore the neurobiological underpinnings of his behavioral pathology.

A multimodal approach using 18F-fluorodeoxyglucose (FDG)-PET, diffusion tensor imaging (DTI), and volumetric MRI was employed, with results compared against healthy controls obtained from the Functional Biomedical Informatics Research Network (FBIRN) data repository (n = 16 for PET; n = 42 for DTI/MRI). FDG-PET revealed reduced neocortex-to-cerebellum ratios consistent with FASD, alongside hypermetabolism in the cerebellum, posterior cingulate, and superior temporal gyrus, suggesting limbic irritability. DTI demonstrated reduced fractional anisotropy in major white-matter tracts implicated in executive function, while volumetric MRI revealed enlargement of the right putamen and increased relative volumes in the hippocampus and amygdala.

Findings suggest that FASD-related abnormalities, compounded by head trauma and early adversity, may contribute to violent behavior. This case underscores the forensic value of integrating PET, DTI, and MRI to inform neuropsychiatric evaluation and risk assessment in high-risk offenders.

## Introduction

Fetal alcohol spectrum disorder (FASD) is a condition that arises from prenatal exposure to alcohol. This prenatal exposure can disrupt critical pathways in brain development, resulting in lasting structural and functional abnormalities [1]. One of the brain regions that is affected most is the prefrontal cortex, which plays a central role in executive functions such as decision-making, emotional regulation, and impulse control [2]. Damage to this region can lead to a condition called orbitofrontal cortex syndrome, which is characterized by poor judgment, impulsivity, and socially inappropriate behavior [3-4]. A specific subregion within the prefrontal cortex, the orbitofrontal cortex, appears to be especially vulnerable to prenatal alcohol exposure, and studies have shown decreased function and volume in this region among individuals with FASD [5].

In this report, we aim to characterize the neurobiological underpinnings of severe behavioral dysregulation in a patient with FASD using multimodal neuroimaging techniques. While FASD is a known risk factor for behavioral dysregulation, the precise mechanisms linking it to extreme behaviors, such as violent crime, remain unclear. Neuroimaging research suggests that prenatal alcohol exposure disrupts the development of neural pathways involved in emotional and behavioral regulation; however, mounting evidence suggests that FASD rarely acts alone. Additional factors, such as traumatic brain injury (TBI) and adverse childhood experiences (ACEs), may compound underlying vulnerabilities and worsen behavioral outcomes [6]. For example, studies have linked ACEs to long-term changes in hippocampal and amygdala volumes, regions associated with emotional memory, fear, and threat perception. TBI has likewise been shown to intensify executive function deficits in individuals with neurodevelopmental conditions such as FASD.

Multimodal neuroimaging provides a framework for assessing both functional and structural brain abnormalities. 18F-fluorodeoxyglucose (FDG)-PET measures regional cerebral glucose metabolism as an indicator of neuronal activity, while diffusion tensor imaging (DTI) evaluates white matter integrity by quantifying the directional diffusion of water within neural tracts. Together, these techniques allow for a more comprehensive assessment of brain dysfunction than either modality alone.

This interaction between biological vulnerability and psychosocial adversity is consistent with a biopsychosocial model of violence, which emphasizes the convergence of early trauma, neurological dysfunction, and the social environment in the development of antisocial behavior [7-8].

This study documents the case of a 35-year-old subject with orbitofrontal cortex dysfunction and executive function deficits who committed a violent crime following an emotional trigger. Using 18F-FDG PET and DTI, we explored the neurobiological underpinnings of his behavior in the context of FASD, ACEs, and TBI. Our goal is to contribute to the growing literature on FASD-related impairments and discuss their implications for both clinical and forensic settings.

## Case presentation

In mid-2019, a male inmate with a longstanding history of severe neuropsychiatric illness and recurrent violent behavior was referred for forensic neuroimaging evaluation while incarcerated in a state correctional facility. At the time of evaluation, he was serving multiple life sentences related to prior violent offenses. The subject had a complex psychiatric and neurodevelopmental history, including diagnoses of major depressive disorder, bipolar disorder, schizophrenia, post-traumatic stress disorder (PTSD), FASD, and orbitofrontal cortex syndrome. Clinically, these conditions were associated with marked impairments in impulse control, emotional regulation, and executive functioning. Prior forensic neuropsychiatric and neurological assessments documented profound behavioral dysregulation and recommended neuroimaging to further characterize underlying neural abnormalities. Formal neuropsychiatric evaluations consistently demonstrated deficits in executive function, impulse control, and emotional regulation. Overall, the subject exhibited a clinical phenotype characterized by severe executive dysfunction, impaired impulse control, emotional dysregulation, and recurrent violent behavior in the setting of FASD, TBI, and significant early-life trauma. These symptoms were present from early adolescence and progressively worsened into adulthood, culminating in repeated violent offenses.

The subject’s developmental history was notable for extreme early-life adversity. He was born to a mother with severe mental illness who reportedly consumed alcohol during pregnancy and received inadequate prenatal care, increasing the likelihood of FASD. Following maternal death in childhood, the subject experienced prolonged instability across foster care and custodial placements, with chronic exposure to physical, sexual, and emotional abuse, as well as severe neglect. He sustained multiple TBIs throughout childhood and adolescence, including episodes involving loss of consciousness related to interpersonal violence and institutional restraint. Consistent with this history, the subject scored 8 out of 10 on the ACEs scale, placing him among the highest-risk strata for adverse neuropsychiatric and behavioral outcomes [9]. Given the convergence of FASD, repeated traumatic brain injury, extreme early-life trauma, and persistent behavioral dysregulation, multimodal neuroimaging was undertaken to assess structural and functional abnormalities potentially underlying this clinical phenotype. 

The subject underwent 18F-FDG-PET imaging in April 2022. Image processing and statistical analysis were performed using Statistical Parametric Mapping (SPM; Wellcome Centre for Human Neuroimaging, University College London, UK) and VINCI software (Max Planck Institute for Neurological Research, Cologne, Germany), with normalization to Montreal Neurological Institute (MNI) standard space [10] and comparisons made to age- and sex-matched controls. Z-map analysis was performed to identify regions of significant metabolic deviation compared with controls. Statistical comparisons were performed using one-sample Z-tests to assess deviation of the subject’s imaging values from a normative control distribution. This approach is commonly used in single-subject neuroimaging analyses to identify clinically meaningful departures from reference populations.

Given the single-subject design and relatively small control sample (n = 16 for PET), statistical findings should be interpreted with caution. A voxel-wise threshold of p < 0.01 (uncorrected) was used to identify regions of potential interest, consistent with exploratory analyses in single-case neuroimaging studies. However, no formal correction for multiple comparisons (e.g., false discovery rate or Bonferroni correction) was applied, which increases the risk of type I error. As such, these findings should be considered descriptive and hypothesis-generating rather than definitive.

**Figure 1:**
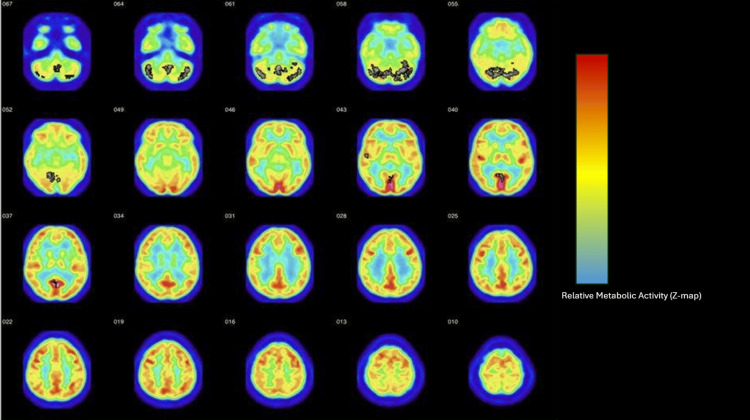
Z-map analysis of hypermetabolism in the 35-year-old subject’s fluorodeoxyglucose (FDG)-PET scan. Transaxial FDG-PET Z-map slices illustrating regions of significant metabolic deviation in the subject relative to an age- and sex-matched control group derived from the Functional Biomedical Informatics Research Network (FBIRN) dataset. The colorbar represents the Z-score deviation from the control mean (warmer colors indicate hypermetabolism, cooler colors indicate hypometabolism). The numbers displayed next to each image represent axial slice coordinates in the Montreal Neurological Institute (MNI) space (mm). Statistical parametric mapping (SPM) was performed using a threshold of p < 0.01 (uncorrected) with a minimum cluster size of 30 voxels.

**Figure 2:**
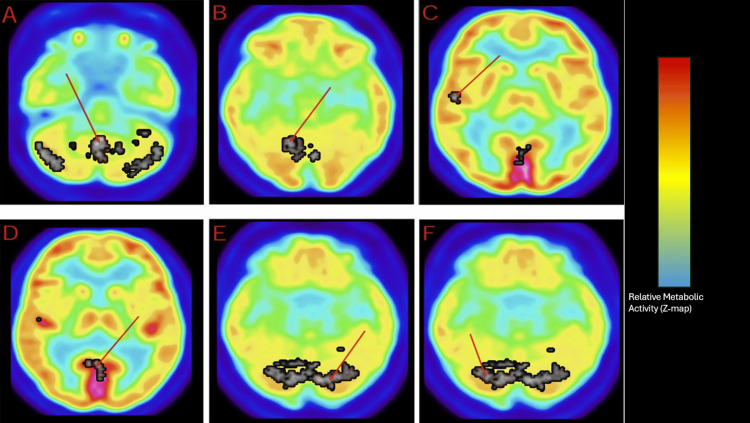
Hypermetabolic regions of interest in the 35-year-old subject’s fluorodeoxyglucose (FDG)-PET scan. Regional FDG-PET Z-map overlays illustrating areas of significant metabolic deviation in the subject relative to an age- and sex-matched control group from the Functional Biomedical Informatics Research Network (FBIRN) dataset used as the reference for Z-map analysis. The colorbar represents Z-score deviation from the control mean (warmer colors indicate hypermetabolism, cooler colors indicate hypometabolism). Panels A–F depict representative axial slices highlighting regions of interest. The red arrows indicate focal regions of increased metabolic activity identified on the Z-map. Statistical parametric mapping (SPM) was performed using a threshold of p < 0.01 (uncorrected) with a minimum cluster size of 30 voxels (n = number of contiguous voxels within a cluster).

Key imaging findings are summarized below, with detailed regional analyses provided in the accompanying figures and tables. The FDG-PET analysis revealed metabolic abnormalities in the subject. These results were compared to 16 healthy controls obtained from the Functional Biomedical Informatics Research Network (FBIRN) repository [11]. Whole-lobe analyses showed hypermetabolism in the cerebellum and hypometabolism in the neocortex-to-cerebellum ratio. Hypermetabolic regions included the cerebellum, posterior lobe, and uvula region; the right cerebellum, posterior lobe, declive region, and the right superior temporal gyrus, Brodmann area 22; posterior cingulate, Brodmann area 30; the left cerebellum, posterior lobe, pyramis region; and the right cerebellum, anterior lobe, culmen. These results are visualized using a Z-map showing regions of hypermetabolism in the brain (Figure 1). The specific regions of hypermetabolism identified in the PET scan are further illustrated in regional overlays (Figure 2). No statistically significant differences were detected in the neocortex, frontal lobe, occipital lobe, or the frontal-to-occipital lobe ratio. A summary of the regional and whole-lobe metabolic findings is presented in Tables 1-2. These metabolic abnormalities are consistent with disruptions in neural circuits underlying executive function, emotional regulation, and impulse control, aligning with the subject’s observed behavioral phenotype.

**Table 1:** Fluorodeoxyglucose (FDG)-PET regional regions of interest (ROI) analysis. Case Subject represents the index patient. Average and STD represent the mean and standard deviation of the control group (n = 16, Functional Biomedical Informatics Research Network (FBIRN) dataset). Values reflect relative glucose metabolism measured via FDG-PET (standardized uptake ratios). Statistical comparisons were performed using a one-sample Z-test. X, Y, and Z coordinates represent Talairach space locations of identified regions. P-values indicate statistical significance.

FDG-PET Regional ROI Analysis	Case Subject	Average	STD	Z-score	P-value	X	Y	Z
A) Cerebellum, Posterior Lobe, Uvula	1.33	0.98	0.09	3.78	1.6E-04	0	-66	-34
B) Right Cerebellum, Posterior Lobe, Declive	1.21	0.97	0.08	3.01	2.7E-03	12	-56	-16
C) Right Superior Temporal Gyrus, Brodmann area 22	1.57	1.19	0.14	2.63	8.6E-03	56	-8	2
D) Posterior Cingulate, Brodmann area 30	1.77	1.35	0.15	2.80	5.1E-03	4	-56	8
E) Left Cerebellum, Posterior Lobe, Pyramis	1.34	1.10	0.11	2.31	2.1E-02	-24	-66	-28
F) Right Cerebellum, Anterior Lobe, Culmen	1.33	1.07	0.08	3.17	1.5E-03	26	-60	-22

**Table 2:** Fluorodeoxyglucose (FDG)-PET whole lobe regions of interest (ROI) analysis. Case Subject represents the index patient. Average and STD represent the mean and standard deviation of the control group (n = 16, Functional Biomedical Informatics Research Network (FBIRN) dataset). Values reflect relative glucose metabolism measured via FDG-PET (standardized uptake ratios). Statistical comparisons were performed using a one-sample Z-test. P-values indicate statistical significance.

FDG-PET Whole Lobe ROI Analysis	Case Subject	Average	STD	Z-score	P-value
Neocortex	0.99	0.99	0.02	-0.28	7.8E-01
Cerebellum	1.15	0.97	0.07	2.56	0.01
Neocortex/Cerebellum Ratio	0.86	1.03	0.07	-2.36	1.8E-02
Frontal Lobe	1.02	1.08	0.05	-1.03	0.30
Occipital Lobe	1.17	1.12	0.06	0.76	0.45
Frontal/ Occipital Lobe Ratio	0.88	0.96	0.07	-1.23	2.2E-01

The Iowa Interview for Partial Seizure-Like Symptoms (IIPSS) was administered after his PET scan [12]. This structured diagnostic tool is designed to evaluate seizure-like symptoms by systematically assessing sensory, cognitive, emotional, and physical experiences commonly associated with partial (focal) seizures. Responses were recorded and scored according to standard IIPSS criteria. The interview involved targeted questions across multiple symptom categories, including questions concerning sensory disturbances, cognitive impairments, mood and emotional fluctuations, and physical or sleep-related symptoms. Each response was assigned a score reflecting the severity and frequency of the reported symptoms. Each item was scored on a standardized ordinal scale reflecting symptom frequency, and individual item scores were summed to generate a total IIPSS score, with higher scores indicating greater severity and frequency of seizure-like symptoms across sensory, cognitive, emotional, and physical domains. The normative dataset, derived from the normative sample for the 40-item version of the IIPSS (n = 115), provides percentile rankings for each total score, allowing for comparisons between the subject’s results and a neurologically typical population. The subject scored a total of 74 on the IIPSS, placing him above the 99^th^ percentile relative to normative data (n = 115). He reported frequent symptoms across sensory, cognitive, mood, and physical domains, including episodic sensory disturbances, cognitive impairments, mood dysregulation, and sleep-related abnormalities.

MRI DTI scans were obtained in April 2022, using a 3 Tesla MRI scanner (GE Medical Systems, Chicago, Illinois, USA). DTI images were preprocessed and normalized using the Functional MRI of the Brain Software Library (FSL; Oxford Centre for Functional MRI of the Brain, University of Oxford, Oxford, UK). The scans were compared to 42 age- and sex-matched neurologically normal controls obtained from the FBIRN data repository [11]. To assess white matter abnormalities, positive and negative Z-maps were generated via SPM software. Fractional anisotropy (FA) values were analyzed in regions of interest (ROIs) and whole-brain metrics using the VINCI software, with comparisons made to a subset of 15 controls more closely matched by sex and age for DTI.

**Figure 3:**
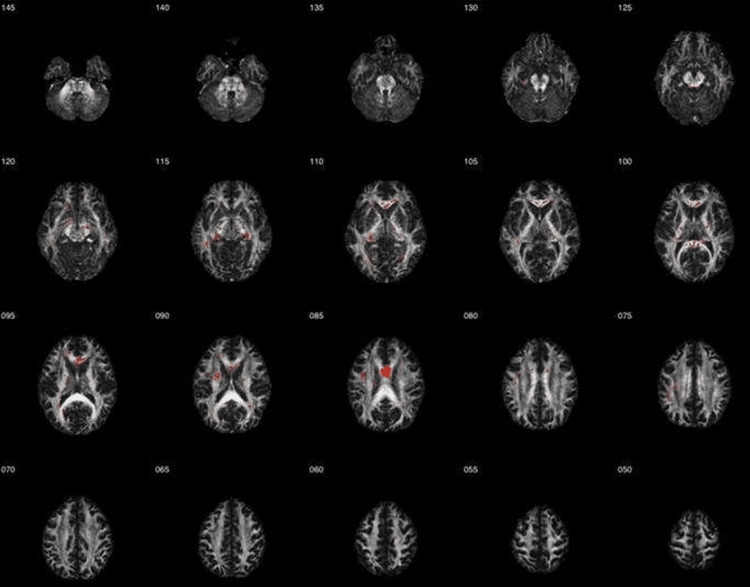
Diffusion tensor imaging (DTI) Z-map indicating reduced fractional anisotropy (FA) Axial DTI Z-map slices illustrating white matter abnormalities in the subject relative to an age- and sex-matched control group derived from the Functional Biomedical Informatics Research Network (FBIRN) dataset. The numbers displayed next to each image represent axial slice coordinates in Montreal Neurological Institute (MNI) space (mm). Regions highlighted in red indicate areas of reduced FA compared with controls, reflecting white matter disruption.

**Figure 4:**
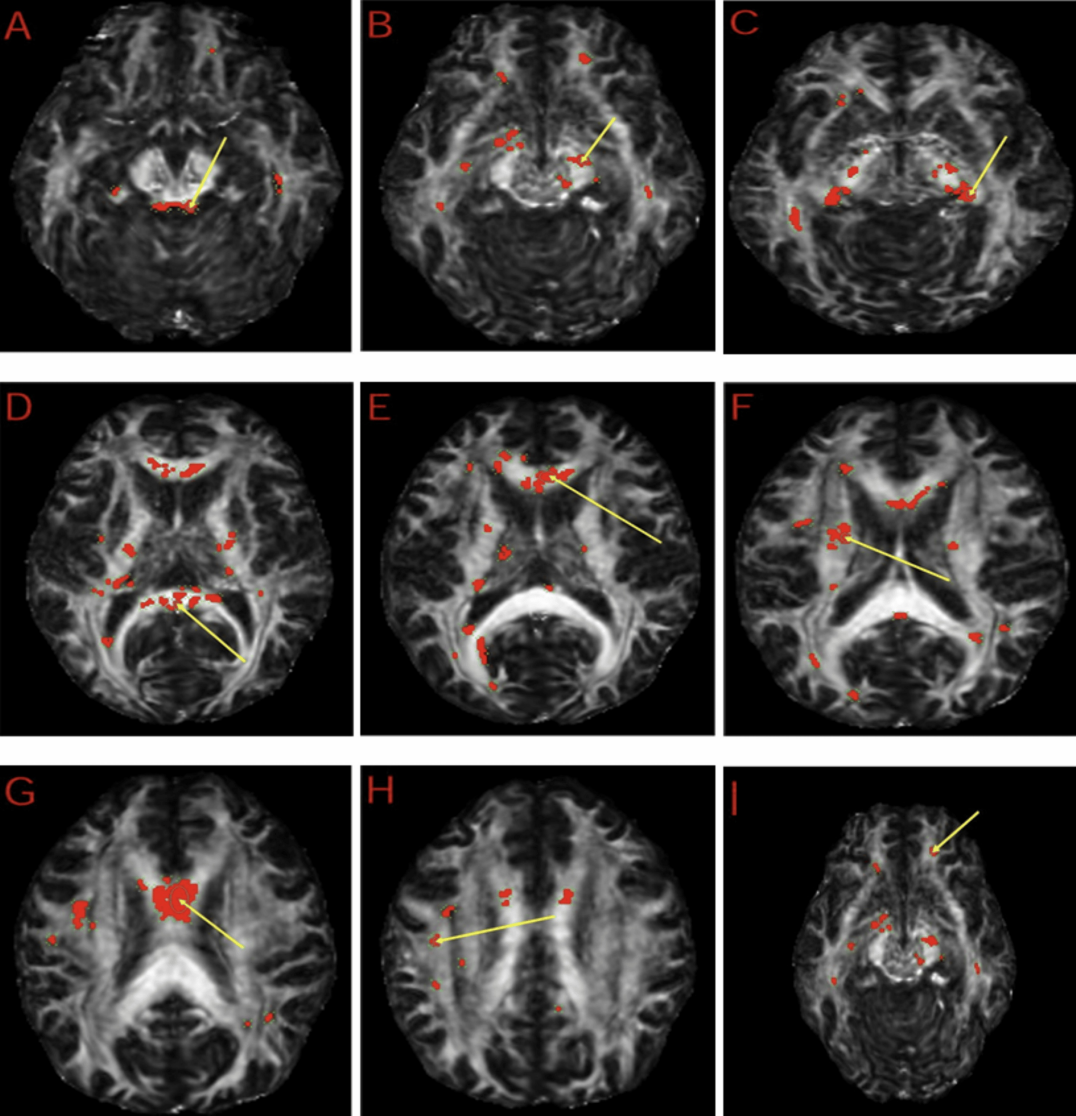
Regions of interest (ROIs) with reduced fractional anisotropy (FA) from diffusion tensor imaging (DTI). DTI analysis identifying ROIs with significant reductions in FA. Regions highlighted in red indicate areas of reduced fractional anisotropy, reflecting white matter disruption. The yellow arrows indicate representative focal regions of reduced FA identified on the Z-map. Affected regions include the superior cerebellar peduncle (A), cerebral peduncle (B), fornix/stria terminalis (C, H), corpus callosum (D, E, G), and the anterior limb of the internal capsule (F). These findings are consistent with disruptions in white matter pathways involved in executive function, impulse control, and emotional regulation.

**Figure 5:**
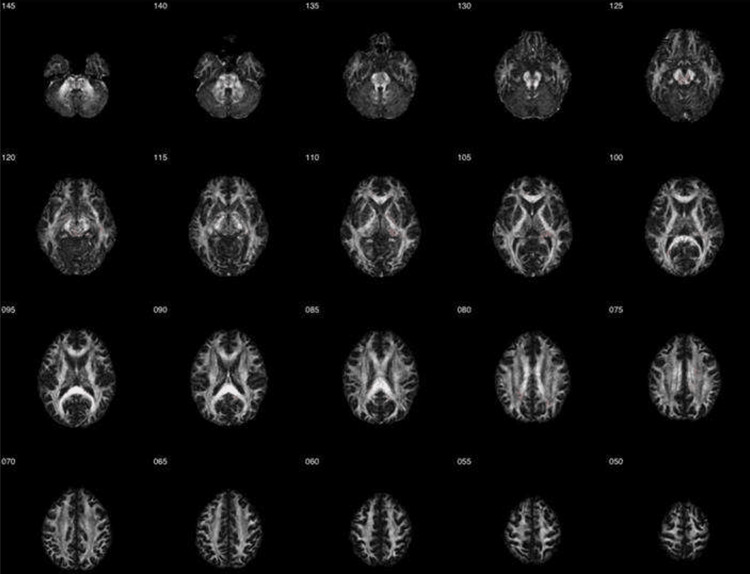
Diffusion tensor imaging (DTI) Z-map indicating increased fractional anisotropy (FA) Axial DTI Z-map slices illustrating areas of increased FA in the subject relative to an age- and sex-matched control group. The numbers displayed next to each image represent axial slice coordinates in the Montreal Neurological Institute (MNI) space (mm).

**Figure 6:**
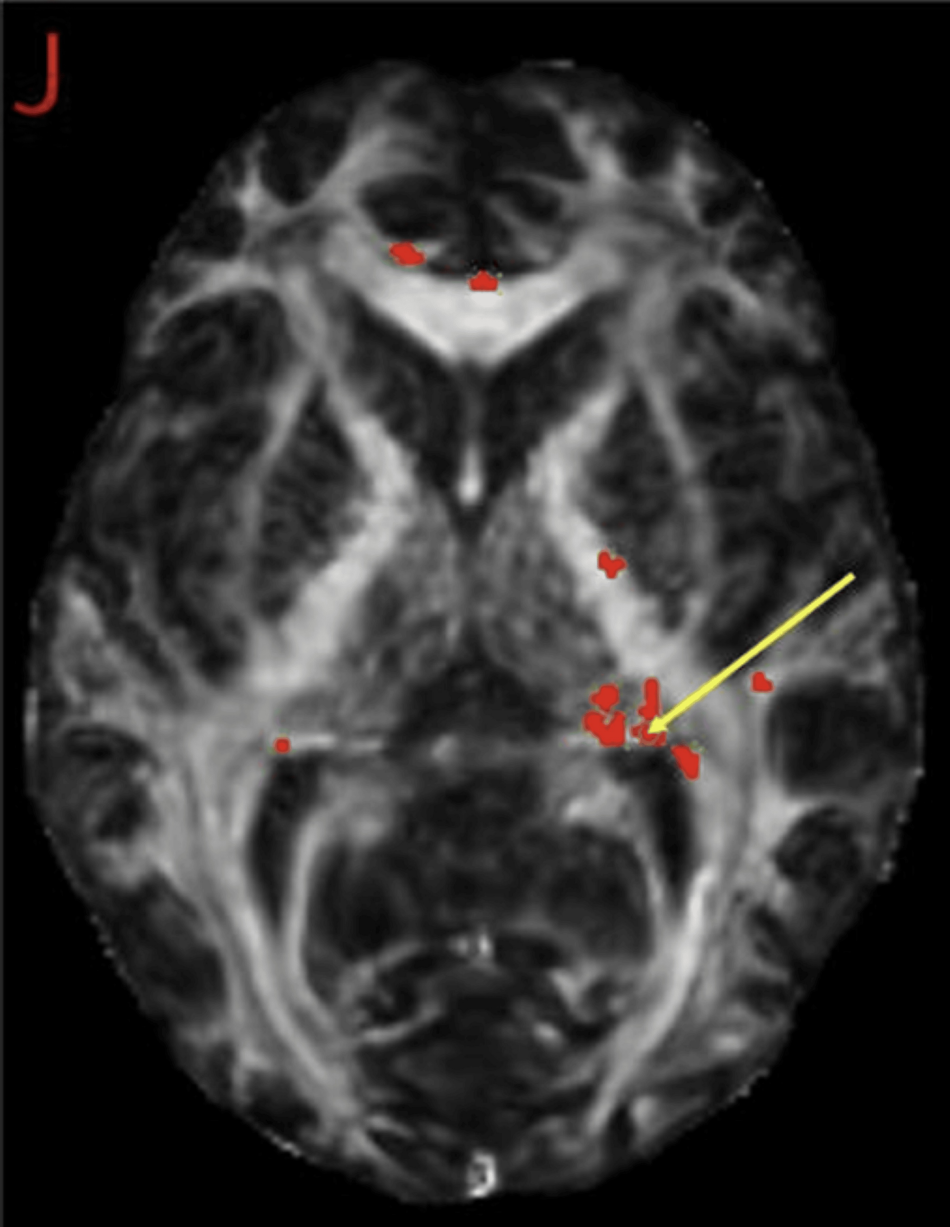
Diffusion tensor imaging (DTI) and positive region of interest (ROI) analysis. DTI analysis showing a significant region of increased fractional anisotropy (FA) in the fornix (crus)/stria terminalis (highlighted in red). The yellow arrow indicates a focal region of increased fractional anisotropy within the fornix/stria terminalis complex. This elevated FA may reflect compensatory mechanisms or altered structural connectivity associated with traumatic brain injury (TBI) or early life adverse experiences.

MRI DTI revealed white matter abnormalities. These results were compared to 42 healthy controls obtained from the FBIRN repository. Regions with reduced FA included the right brainstem, midbrain, superior cerebellar peduncle, right fornix (crus)/stria terminalis, corpus callosum, left anterior limb of the internal capsule, left precentral gyrus, and the superior longitudinal fasciculus. These areas of reduced white matter integrity are visualized in the DTI Z-map, highlighting regions of decreased FA, along with their respective ROI analysis (Figures 3-4). Regions of increased FA were also found in the right fornix (crus)/stria terminalis. These areas of increased white matter integrity are visualized in the DTI Z-map, highlighting regions of increased fractional anisotropy, along with their respective ROI analysis (Figures 5-6). No statistically significant differences were observed in other regions of interest. A summary of the regional metabolic findings is presented in Table 3.

**Table 3:** Diffusion tensor imaging (DTI) Analysis. Fractional anisotropy (FA) values for selected regions of interest (ROIs) in a 35-year-old subject’s brain compared to normative controls (n = 42). Z-scores reflect the standardized deviation from the control group mean, and p-values indicate statistical significance. Statistical comparisons were performed using a one-sample Z-test. Negative Z-scores denote reduced FA, while positive Z-scores indicate increased FA. Talairach coordinates (X, Y, Z) identify the precise anatomical location of each ROI.

DTI Regional ROI Analysis	Subject	Average	STD	Z-score	P-value	X	Y	Z
A) Right Superior Cerebellar Peduncle	0.26	0.49	0.06	-3.55	3.8E-04	6	-43	-27
B) Cerebral Peduncle	0.43	0.59	0.03	-4.95	7.5E-07	17	-11	-11
C) Fornix (Crus)/Stria Terminalis	0.43	0.65	0.05	-4.80	1.6E-06	26	-23	-7
D) Posterior Corpus Callosum	0.56	0.73	0.06	-2.62	8.8E-03	0	-37	9
E) Anterior Corpus Callosum	0.64	0.73	0.04	-2.13	3.4E-02	1	28	5
F) Left Anterior Limb of Internal Capsule	0.49	0.59	0.05	-2.13	3.3E-02	-17	2	17
G) Corpus Callosum	0.55	0.67	0.05	-2.38	1.7E-02	-2	3	21
H) Precentral Gyrus, Superior Longitudinal Fasciculus	0.51	0.59	0.04	-2.15	3.1E-02	-44	-15	33
I) Frontal Lobe, Inferior fronto-occipital Fasciculus Right	0.40	0.53	0.05	-2.63	8.5E-03	19	40	-7
J) Fornix /Stria Terminalis	0.87	0.50	0.08	4.67	3.1E-06	23	-31	-1

NeuroQuant analysis (NeuroQuant; Cortechs.ai, San Diego, CA, USA) revealed significant volumetric differences in the subject compared to controls. Regions with increased absolute volume included the right putamen, which showed the most significant increase (p = 0.031). Furthermore, relative volume analyses (adjusted for intracranial volume) revealed significant increases in the left and right forebrain parenchyma, right cortical gray matter, left hippocampus, left amygdala, right amygdala, left putamen, and right putamen, with the right putamen showing the highest Z-score (2.80, p = 0.0051). No significant differences were observed in the caudate, pallidum, thalamus, or cerebellum. Whole-brain analyses demonstrated a notable increase in total forebrain parenchyma volume, whereas ventricular volumes remained within normal limits. These findings indicate structural abnormalities in subcortical gray matter regions associated with motor function, learning, and emotional processing. A summary of absolute and relative volumetric findings is presented in Tables 4-5, respectively.

**Table 4:** Absolute brain volume abnormalities Case Subject represents the index patient. Mean and SD represent the mean and standard deviation of the control group (n = 42, Functional Biomedical Informatics Research Network (FBIRN) dataset). Values reflect regional brain volumes measured via volumetric MRI (NeuroQuant) in cubic centimeters (cm³). Statistical comparisons were performed using a one-sample Z-test. P-values indicate statistical significance.

Absolute Volume (cm^3^)	Subject	Mean	SD	Z-score	P-value
Left Forebrain Parenchyma	586.30	573.79	57.17	0.22	8.3E-01
Right Forebrain Parenchyma	598.75	581.10	59.03	0.30	7.6E-01
Left-Right Forebrain Parenchyma	-12.45	-7.30	7.35	-0.70	4.8E-01
Left Cortical Gray Matter	303.64	276.44	38.36	0.71	4.8E-01
Right Cortical Gray Matter	316.56	280.67	38.45	0.93	3.5E-01
Left-Right Cortical Gray Matter	-12.92	-4.22	5.65	-1.54	1.2E-01
Left Lateral Ventricle	6.93	9.26	3.52	-0.66	5.1E-01
Right Lateral Ventricle	7.47	8.73	3.49	-0.36	7.2E-01
Left-Right Lateral Ventricle	-0.54	0.53	2.28	-0.47	6.4E-01
Left Inferior Lateral Ventricle	0.92	0.98	0.26	-0.24	8.1E-01
Right Inferior Lateral Ventricle	0.36	0.93	0.32	-1.77	7.7E-02
Left-Right Inferior Lateral Ventricle	0.56	0.05	0.35	1.45	1.5E-01
Left Hippocampus	4.44	4.09	0.41	0.85	4.0E-01
Right Hippocampus	3.91	4.20	0.53	-0.55	5.8E-01
Left-Right Hippocampus	0.53	-0.11	0.43	1.46	1.4E-01
Left Amygdala	2.20	1.91	0.26	1.13	2.6E-01
Right Amygdala	2.15	1.91	0.28	0.88	3.8E-01
Left-Right Amygdala	0.05	0.00	0.13	0.38	7.0E-01
Left Caudate	3.00	3.60	0.65	-0.92	3.6E-01
Right Caudate	3.22	3.78	0.72	-0.78	4.4E-01
Left-Right Caudate	-0.22	-0.19	0.50	-0.07	9.4E-01
Left Putamen	6.86	5.75	0.63	1.75	8.0E-02
Right Putamen	6.55	5.35	0.56	2.16	3.1E-02
Left-Right Putamen	0.31	0.40	0.36	-0.24	8.1E-01
Left Pallidum	0.84	1.06	0.17	-1.34	1.8E-01
Right Pallidum	0.86	1.13	0.19	-1.40	1.6E-01
Left-Right Pallidum	-0.02	-0.06	0.14	0.31	7.6E-01
Left Thalamus	8.92	8.62	1.00	0.30	7.6E-01
Right Thalamus	8.31	9.90	1.71	-0.93	3.5E-01
Left-Right Thalamus	0.61	-1.28	1.17	1.62	1.1E-01
Left Cerebellum	76.69	75.50	8.18	0.15	8.8E-01
Right Cerebellum	75.40	74.55	8.03	0.11	9.1E-01
Left-Right Cerebellum	1.29	0.95	2.47	0.14	8.9E-01
Intracranial Volume	1634.39	1710.27	152.45	-0.50	6.2E-01

**Table 5:** Relative brain volume abnormalities Case Subject represents the index patient. Mean and SD represent the mean and standard deviation of the control group (n = 42, Functional Biomedical Informatics Research Network (FBIRN) dataset). Values reflect relative brain volumes expressed as a percentage of intracranial volume (%ICV), derived from volumetric MRI (NeuroQuant). Statistical comparisons were performed using a one-sample Z-test. P-values indicate statistical significance. ICV: intracranial volume

Relative Volume (% ICV)	Subject	Mean	SD	Z-score	P-value
Left Forebrain Parenchyma	37.05	33.43	1.13	3.21	1.3E-03
Right Forebrain Parenchyma	37.83	33.85	1.26	3.14	1.7E-03
Left-Right Forebrain Parenchyma	-0.78	-0.42	0.42	-0.86	3.9E-01
Left Cortical Gray Matter	19.19	16.42	2.46	1.13	2.6E-01
Right Cortical Gray Matter	20.00	16.36	1.70	2.14	3.2E-02
Left-Right Cortical Gray Matter	-0.81	0.06	1.80	-0.48	6.3E-01
Left Lateral Ventricle	0.44	0.54	0.21	-0.49	6.2E-01
Right Lateral Ventricle	0.47	0.51	0.20	-0.22	8.3E-01
Left-Right Lateral Ventricle	-0.03	0.03	0.13	-0.45	6.5E-01
Left Inferior Lateral Ventricle	0.06	0.06	0.01	0.26	7.9E-01
Right Inferior Lateral Ventricle	0.02	0.05	0.02	-1.86	6.3E-02
Left-Right Inferior Lateral Ventricle	0.04	0.00	0.02	1.88	6.0E-02
Left Hippocampus	0.28	0.24	0.02	2.04	4.1E-02
Right Hippocampus	0.25	0.25	0.03	0.16	8.7E-01
Left-Right Hippocampus	0.03	-0.01	0.02	1.49	1.4E-01
Left Amygdala	0.14	0.11	0.01	2.37	1.8E-02
Right Amygdala	0.14	0.11	0.01	2.15	3.2E-02
Left-Right Amygdala	0.00	0.00	0.01	-0.04	9.7E-01
Left Caudate	0.19	0.21	0.04	-0.53	6.0E-01
Right Caudate	0.20	0.22	0.03	-0.64	5.2E-01
Left-Right Caudate	-0.01	-0.01	0.03	0.03	9.8E-01
Left Putamen	0.43	0.34	0.04	2.21	2.7E-02
Right Putamen	0.41	0.31	0.03	2.80	5.1E-03
Left-Right Putamen	0.02	0.02	0.02	-0.22	8.3E-01
Left Pallidum	0.05	0.06	0.01	-1.23	2.2E-01
Right Pallidum	0.05	0.07	0.01	-1.40	1.6E-01
Left-Right Pallidum	0.00	0.00	0.01	0.50	6.2E-01
Left Thalamus	0.56	0.51	0.06	0.82	4.1E-01
Right Thalamus	0.53	0.58	0.08	-0.55	5.8E-01
Left-Right Thalamus	0.03	-0.07	0.07	1.39	1.6E-01
Left Cerebellum	4.85	4.41	0.38	1.16	2.5E-01
Right Cerebellum	4.76	4.35	0.38	1.06	2.9E-01
Left-Right Cerebellum	0.09	0.05	0.14	0.26	7.9E-01

Volumetric asymmetry analysis via NeuroQuant morphometry revealed multiple lateralized abnormalities across key brain structures. The most pronounced asymmetry was observed in the inferior lateral ventricles, which demonstrated an 86.56% asymmetry index, with the left ventricle volume (0.92 cm³) being significantly larger than the right ventricle volume (0.36 cm³). These morphometric asymmetries may reflect underlying neurodevelopmental or neurotoxic processes consistent with the subject’s history of FASD, TBI, and ACEs. 

## Discussion

This study highlights significant findings regarding FASD in conjunction with TBI and ACEs and their potential contribution to impulse control deficits and violent behavior, with a particular focus on the decreased neocortical to cerebellar ratio. Much like in prior research studies, our findings show that the orbitofrontal cortex, a critical region for impulse control and emotional regulation, is modestly affected in FASD patients [13]. The FDG-PET imaging of the subject revealed a decreased neocortex-to-cerebellum metabolic ratio, indicating diffuse neocortical hypometabolism. Given that the orbitofrontal cortex is part of the neocortex, this finding further supports the presence of impaired metabolic activity in this region. This finding aligns with existing evidence linking prefrontal dysfunction, especially in the orbitofrontal cortex, to impulsivity and aggression and expands our understanding of how diffused cortical deficits in FASD patients may contribute to violent behavior.

In addition to neocortical hypometabolism, FDG-PET revealed hypermetabolism in the right superior temporal gyrus and posterior cingulate, which are regions associated with limbic processing. These abnormalities may reflect limbic kindling effects tied to chronic stress and adverse childhood experiences, which are known to sensitize the brain's stress response systems over time [14]. Furthermore, hypermetabolism observed in the cerebellum, including the declive and culmen, aligns with prior findings in individuals with FASD, where cerebellar abnormalities are thought to reflect disrupted sensorimotor integration and compensatory mechanisms [5,15]. Taken together, these metabolic alterations reinforce the role of both neurodevelopmental insult and environmental adversity in shaping the neural circuits underlying behavioral dysregulation.

In addition to FDG-PET imaging, DTI findings revealed significant structural abnormalities in key white matter pathways associated with executive function, impulse control, and emotional regulation. The analysis revealed reduced FA in multiple regions, including the corpus callosum, superior cerebellar peduncle, and fornix, tracts known to underlie cognitive and behavioral regulation. Notably, decreased FA in the superior cerebellar peduncle suggests disrupted connectivity within cerebellar-thalamo-cortical circuits. This is supported by evidence linking lower FA in the superior cerebellar peduncles to poorer working memory performance, particularly in tasks requiring executive processing such as the two-back task [16]. These findings align with the subject’s pronounced deficits in executive control and behavioral inhibition. Similarly, reduced FA in the corpus callosum suggests impaired interhemispheric integration. Additionally, the corpus callosum is one of the most frequently affected regions in TBI, with decreased FA commonly observed as a marker of diffuse axonal injury [17]. Taken together, these findings suggest that the subject’s white matter abnormalities may reflect the cumulative effects of both prenatal alcohol exposure and subsequent head trauma.

Structural abnormalities in the fornix, a key tract supporting memory and emotional regulation, further support dysregulated affective processing [18]. Interestingly, increased FA in the right fornix/stria terminalis may indicate compensatory hyperconnectivity, a phenomenon sometimes observed in individuals with early neurodevelopmental insults [19]. This interpretation remains speculative and should be interpreted with caution. Some studies suggest that increased FA in the fornix and other limbic tracts may be more closely associated with TBI and ACEs than with FASD alone. A 2021 study revealed a multidimensional MRI signature of diffuse axonal injury in TBI patients, which included increased FA in certain white matter regions, possibly reflecting axonal compression or reorganization [20]. Another study demonstrated that FA was altered in the fornix among individuals with a history of early life adversity, linking limbic white matter changes more strongly to stress-related exposures than to prenatal alcohol exposure. Considering these findings, the elevated FA observed in the subject’s right fornix may be better interpreted because of repeated trauma and adverse childhood experiences, rather than a direct outcome of FASD. Collectively, these findings reinforce the interpretation that the subject’s neurocognitive impairments have a structural basis, which is consistent with his history of impulsivity and violent behavior.

NeuroQuant analysis also revealed increased volumes in regions such as the amygdala and hippocampus. These findings are supported by prior research linking the abnormal enlargement of these structures to conditions such as temporal lobe epilepsy [21], posttraumatic stress disorder, and disorganized attachment stemming from early adversity. In these contexts, amygdala hyperactivity and increased volume have been associated with heightened emotional reactivity and impaired regulation, traits that could underlie the behavioral dysregulation observed in this case.

In addition to these limbic structures, volumetric analysis also revealed enlargement of the putamen. This finding aligns with prior neuroimaging studies across several psychiatric conditions, suggesting that putamen enlargement may be a transdiagnostic marker of dysregulated cognitive and affective control. For example, individuals with PTSD, particularly those with dissociative subtypes, exhibit larger putamen volumes, which positively correlate with dissociative symptom severity [22]. These findings suggest that putamen enlargement may reflect aberrant development or activity within cortico-striatal circuits involved in behavioral regulation, a mechanism that may underlie the subject's disinhibited and impulsive conduct.

Volumetric morphometry analysis revealed asymmetries in the inferior lateral ventricles (86.56%). While these findings are visually notable, they did not meet standard thresholds for statistical significance and should be interpreted with caution. At present, there is insufficient evidence to draw firm conclusions about the clinical relevance of these asymmetries. However, prior neuroimaging research has reported that subtle structural asymmetries may sometimes arise in individuals with histories of early neurodevelopmental adversity, such as prenatal alcohol exposure or childhood trauma [23]. In this context, these findings may warrant further investigation in larger, more controlled studies.

Another important aspect worth investigating pertains to head trauma. Preliminary evidence suggests that TBI may exacerbate certain neurocognitive challenges in individuals with FASD, though further research is needed to clarify the extent and consistency of these effects. Furthermore, landmark forensic psychiatry studies by Lewis and colleagues [7-8] revealed that violent delinquents frequently share a triad of early neurological impairments, significant childhood trauma, and histories of head injuries. These findings emphasized a biopsychosocial model of violence and provided early empirical support for the idea that neurodevelopmental vulnerability combined with environmental adversity significantly increases the risk of violent behavior. Similarly, individuals with FASD have significantly higher ACEs scores, increasing their likelihood of experiencing head trauma due to physical abuse, neglect, and unstable home environments [24]. Given the subject’s history of early childhood trauma and FASD, any additional head injuries he sustained may have worsened his ability to regulate behavior, supporting the neuroimaging findings of impulse control deficits in this case. While these findings are supported by observed imaging abnormalities and existing literature, interpretations regarding underlying mechanisms (e.g., compensatory changes) remain hypothesis-generating and should be interpreted with caution.

## Conclusions

This case demonstrates convergent structural and functional abnormalities associated with orbitofrontal dysfunction in the context of FASD, TBI, and ACEs. Multimodal neuroimaging revealed neocortical hypometabolism, white matter disruption, and subcortical volumetric abnormalities, which are consistent with impairments in impulse control, emotional regulation, and behavioral stability.

Although limited by its single-subject design, these findings highlight the potential value of integrated neuroimaging in forensic neuropsychiatric evaluation. Multimodal imaging may provide objective markers of dysfunction that can inform clinical assessment, risk evaluation, and future research into the neurobiological basis of complex behavioral outcomes. However, these findings should be interpreted with caution, as they may not be generalizable to broader populations and are best viewed as hypothesis-generating rather than definitive.
